# The relationship between asthma control and health-related quality of life in asthma and the role of atopy: a cross-sectional study of Nigerian adult asthmatics

**DOI:** 10.11604/pamj.2021.38.393.20625

**Published:** 2021-04-22

**Authors:** Olayemi Fehintola Awopeju, Oluwasina Titus Salami, Adebola Adetiloye, Bamidele Olaiya Adeniyi, Olufemi Olanisun Adewole, Gregory Efosa Erhabor

**Affiliations:** 1 Respiratory Unit, Department of Medicine, Obafemi Awolowo University, Ile Ife, Osun State, Nigeria,; 2 Department of Family Medicine, Obafemi Awolowo University Teaching Hospitals Complex, Ile Ife, Osun State, Nigeria,; 3 Respiratory Unit, Department of Medicine, Federal Medical Centre, Owo, Ondo State, Nigeria

**Keywords:** Adult asthma, atopy, health related quality of life, asthma control test, skin prick test

## Abstract

**Introduction:**

the relationship between asthma control and health-related quality of life (HRQoL) in adult asthmatics is fairly established, but the unique contribution of atopy to this relationship has received less attention. The aim of this study was to quantify the contribution of atopy to this relationship.

**Methods:**

in a cross-sectional study, we assessed HRQoL using mini-Asthma Quality of Life Questionnaire (AQLQ). Asthma control, atopy and lung function were assessed using the Asthma Control Test (ACT), skin prick test and spirometry respectively. Hierarchical multiple regression was used to examine the association between of HRQol and asthma control, atopy and other clinical and demographical factors.

**Results:**

eighty-two adult asthmatics (59 females), with median age of 44 years and median duration of asthma of 15 years were recruited from a tertiary hospital. Fifty-two (63%) were classified as atopic based on sensitization to at least one aeroallergen. The atopic individuals were younger and had better quality of life in activity domain; however, there was no significant difference between the atopic and non-atopic asthmatics in ACT score (19.0 vs 18.0) p=0.91, total AQLQ score (4.9 vs 4.6) p=0.22. The ACT scores correlated positively with total AQLQ scores [rho= 0.53, 95% Confidence Interval (CI) 0.35, 0.67; p< 0.001]. However, atopy contributed significantly to the emotional domain of HRQoL score, p=0.028.

**Conclusion:**

we concluded that better asthma control is associated with better quality of life and atopy contributed uniquely to emotional domain in health-related quality of life.

## Introduction

Asthma causes a huge social impact because it is highly prevalent in many parts of world [1], and it interferes significantly with many aspects of daily life of patients [2]. Health-related quality of life (HRQoL) questionnaires are often used to reflect patients´ perspective and the impact of illness on their daily life [3]. Asthma control has been reported to determine HRQoL [4,5], with correlation coefficient that ranges from (r = 0.49 to 0.78) [6]. Several other factors such as clinical, demographical and psychological factors have been documented to contribute to HRQoL [7,8], besides asthma control, however little data exists on the contribution of atopy to HRQoL in asthmatics. Nevertheless, atopic asthma is the commonest, most widely studied and remains the strongest risk factor for asthma [9,10]. Asthma control and quality of life studies in sub-Saharan Africa have emphasized on certain aspects such as the prevalence, levels of asthma control, quality of life of asthmatics and patient´s related factor [11-15]. However, none assessed the relationship between them controlling for confounders and the influence of atopy. There are also few reports [16-18] on the association of atopy with HRQoL in asthmatics globally, with inconsistent results. For example, Coban and Aydemir reported no relationship between atopy and health related quality of life while another study reported that atopic patients had worse quality of life in patients with nasal polyposis [19]. None of these studies assessed the degree to which HRQoL is predicted by atopy, asthma control and other cofounders such as presence of allergic rhinitis, psychological factors and other clinical factors. To effectively complement pharmacological asthma management, there is a need to understand the how these factors impact on asthma health related quality of life. Therefore, we examined the association of asthma control with asthma specific health related quality of life and explored the contribution of atopy to this association.

## Methods

**Study population:** this was an observational cross-sectional study conducted from December 2015 to November 2016. The patients were previously diagnosed asthmatics, receiving care at the respiratory clinic of Obafemi Awolowo University Teaching Hospitals complex Ile-Ife, Nigeria and presenting for routine clinic care and management. They were consecutively recruited on each clinic day until the sample size was attained. The patients were also stable asthmatics who did not require hospital admission, or emergency room visit. The diagnosis of asthma was based on symptoms and signs consistent with asthma and a respiratory physician´s assessment. In addition, a documented reversibility of FEV1 of > 12% and at least 200 mls in the lung function with the use of 400 mcg salbutamol was also required for the diagnosis of asthma. Inclusion criteria included adult asthmatics who were 18 years and above and must have been diagnosed by a physician in the clinic and must have documented reversibility as stated above. Pregnant ladies were excluded from the study due to variable effects of pregnancy on bronchial asthma and ability to perform lung function test. Subjects with any other concomitant diagnoses such as Chronic Obstructive Pulmonary Disease, bronchiectasis, lung cancer, autoimmune diseases and cardiac illness were excluded. Subjects that have any psychological, physical or language limitation that will prevent the correct completion of the questionnaire were excluded.

A sample of 81 participants was calculated to detect a correlation coefficient of least 0.3 between the asthma control test and health-related quality of life with an alpha of 0.05 (two sided) and power of 80% [20]. Ethical approval was obtained from ethical committee of Obafemi Awolowo University Teaching Hospitals Complex (ERC/2015/12/04). Approved written informed consent was obtained from all subjects before any study related procedures were done. All participants filled questionnaire which included socio-demographics variables, history of the symptoms, duration of symptoms, family history of asthma and history of current drug therapy. Participants were also asked to fill Asthma Control test (ACT) and the Mini Asthma Quality of Life Questionnaire (AQLQ). For the purpose of the present study, General Health Questionnaire 12-item scale (GHQ-12) was chosen as a screening tool for psychological distress. The GHQ scoring method (0-0-1-1) was used, with the sum scores ranging from 0 to 12; participants with the total GHQ-12 score ≤ 2 were considered to have psychological distress [21].

**Asthma control:** asthma control was measured by Asthma Control Test (ACT). ACT is short, simple, self-reporting 5-item questionnaire used to assess asthma symptoms (daytime and nocturnal), use of rescue medications and the effect of asthma on daily functioning at work or home and patients' self-perception of asthma control during the past 4 weeks. Each item includes 5 response options corresponding to a 5-point rating scale. Scores range from 5 to 25, with higher scores indicating better asthma control. It is most widely validated of all asthma control questionnaire [22], and it has test-retest reliability of 0.77 and Cronbach´s Þ of 0.84-0.85 cross-sectionally; 0.79 longitudinally [23].

**Health related of quality of life:** we assessed health related quality of life using the Mini Asthma Quality of Life Questionnaire (AQLQ) with a total of 15 questions in 4 domains (symptoms 5 items, activity limitation 4 items, emotional function 3 items and environmental stimuli 3 items). The answer to each question was scored one to seven (1 = maximum impairment and 7 = no impairment). The domain scores were computed as the mean of domain-specific items and global AQLQ score was computed as the mean of the domain scores. A higher AQLQ score denotes better asthma-related quality of life. The questionnaire is fully validated and has good reliability and is one of the most frequently used instruments to measure asthma related quality of life [24].

**Atopic status:** atopic status was determined by skin prick testing (SPT) performed after discontinuation of antihistamine medications for at least 5 days on the volar aspect of the forearm, using percutaneous multi test method (Multi-Test II Device Lincoln Diagnostics, Decatur, IL, USA) with a panel of aero-allergens common in our environment. This includes the following extract: mould mix (Alternaria alternata, Aureobasidium sorokiniana, Cladosporium sphaerospermum, Drechslera pullulans, Aspergillus niger, and Penicillium notatum), standardized house dust mite mix (Dermatogoides pteronyssinus/farinae), mixed feather (chicken, duck and goose), cockroach (Periplanata americana and Blattella germanica), dog epithelium (Canis familiaris) and 7 standardized grass mix (Timothy, Orchard, June, Redtop, Meadow Fescue, Perennial Rye and Sweet Vernal). Histamine (1.0mg/ml) was used as positive control while glycerinated-saline (NaCl 0.9% glycerin 50%) was used as negative control. All extracts are from ALK ABELLO, Port Washington, New York, USA. A wheal dimension of at least 3mm greater than the negative control was considered to be a positive reaction. Atopy was defined as a positive skin test reaction to at least one of the applied allergens. The sum of the diameters of the reactions to the various antigens was used to measure the intensity of the atopic status.

**Spirometry:** spirometry was performed according to the European Respiratory Society (ERS) and American Thoracic Society (ATS) protocol [25]. Each spirogram was carefully reviewed for acceptability and reproducibility. Only spirometry tests meeting these criteria were included in the analysis. The following were recorded; forced vital capacity (FVC), forced expiratory volume in one second (FEV1), FEV1/FVC, forced expiratory flow between 25% and 75% of FVC (FEF25-75), and peak expiratory flow (PEF). Predicted values were calculated by the reference equations from Africa -American ethnic groups proposed by GLI-2012 [26].

**Statistical analysis:** participants´ characteristics were described using counts and percentages for categorical variables. Normally distributed continuous data are expressed as mean values with standard deviation (SD) and non-normally distributed data are expressed as median values with interquartile ranges (IQR). Normality was tested with Shapiro-Wilk test. To determine the significant differences between asthmatics with and without atopy, unpaired student t-Tests was used for normally distributed continuous variables, Mann-Whitney test for non-normally distributed continuous variables, and chi-square for categorical variables. In addition, we used Spearman's Rank correlation coefficient to determine power and direction of linear relationships between asthma control score and asthma specific quality of life score. Finally, to account for unique contribution of atopy, we used hierarchal linear regression analysis to determine the unique contribution of known factors and atopy to HRQoL. First Step included ACT only, second step included ACT, psychological distress, presence of allergic rhinitis, inhaled corticosteroid usage and % Pred of FEVI while third step included all the factors in second step and atopic status. Each model builds on the prior model by adding a component that is hypothesized to predict asthma-specific HRQoL. The goal of this modelling approach was to show the patterns of change in the regression coefficients as additional variables are included in the models, thus highlighting the unique influence of each component. All reported p-values were two-sided with p < 0.05 considered statistically significant. Statistical analysis was performed with IBM-SPSS software V.20 (IBM, Armonk, New York, USA).

## Results

**Figure 1:**
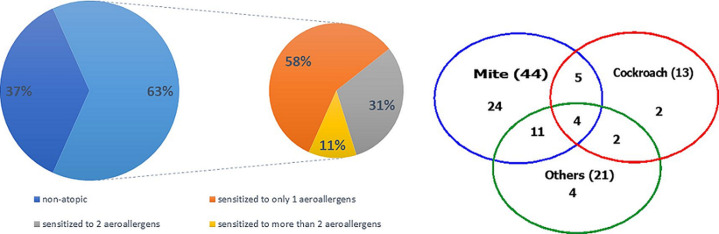
atopic sensitization of the participants

Out of 98 asthmatic patients seen over the study period, sixteen patients were excluded from the analysis due to clinical data incompleteness or refusal to do skin prick test or unavailable spirometry, leaving 82 patients (the median age 44 (29-60) years) in the final analysis group. Fifty-nine (79%) patients were female, and the median duration of symptoms was 15 (8-29) years. The mean of FEV1 percentage predicted was 74.2±28.0. The median (IQR) ACT score was 18.0 (13.0-22.0) and median (IQR) AQLQ score was 4.7 (3.7-5.9). The characteristics of atopic and non-atopic patients were compared in Table 1. Fifty-two of the participants (63%) were classified as atopic based positive skin prick test (SPT) to at least one common aeroallergen. Thirty out of the 52 were sensitized to only one aeroallergen; 16 were sensitized to 2 aeroallergens; 4 were sensitized to 3 aeroallergens while only 2 were sensitized to 4 aeroallergens (Figure 1). The asthmatics were mostly sensitized to house dust mites 44/52 (84.6%), followed by cockroach 13/52 (25%) and then mixed feather 12/52 (23%). The least sensitization was to dog epithelium 2/52 (3.8%). Median number (IQR) of positive SPT and median total diameter (IQR) of wheals were 1 (1-2) and 10 mm (7 mm-16 mm) respectively in the atopic group and none in the non-atopic group. Atopic asthmatics were younger, used less of inhaled corticosteroid and had better quality of life in the activity domain. No statistically significant difference was observed in gender, body mass index, and duration of asthma symptoms, in addition, there was no significant difference between atopic and non-atopic asthmatics in % predicted Forced Expiratory Volume in one second (75 vs 67) p=0.70, ACT score (19 vs 18) p=0.91 and total AQLQ score (4.9 vs 4.6) p=0.24.

**Table 1:** demographics and clinical characteristics of the participants according to atopic status

Variables	Total participants n=82	Non-atopic(n =30)	Atopic(n=52)	†p value
**Age (years)**	44.0(29.0-60.0)	51.5(40.0-60.0)	42.0(24.5-54.5)	0.038*
**BMI (kg/m^2^)**	25.2±5.4	25.16±5.7	24.25±5.3	0.930
**Gender(female)**	59(72%)	20(67%)	39(75%)	0.418
**Duration of symptoms(years)**	15.0(8.0-29.0)	28.5(8.0-39.2)	15.0(9.0-26.0)	0.698
**Highest level of Education**				
Primary	10(12%	7(13%)	3(6%)	0.061*
Secondary	16(19%)	4(13%)	12(23%)	
Post-secondary	56(69%)	19(63%)	37(71%)	
**Emergency visit in the last one year**				
0	55(67%)	19(63%)	36(69%)	
1	18(22%)	7(23%)	11(21%)	0.571
≥2	9(11%)	4(13%)	5(10%)	
**Allergic rhinitis**	30(37%)	7(23%)	23(44%)	0.058
**Inhaled corticosteroid use**	43(52%)	20(67%)	23(44%)	0.048
**GHQ scores ≥ 2**	19(23%)	11(37%)	8(15%)	0.076ï¿½
**Spirometry**				
PEF(L/s)	5.1±2.4	4.8±2.4	5.2±2.4	0.341
PEF(% pred)	59.4±27.1	54.0±28.0	60.8±25.3	0.225
FEV1(L)	2.2±1.3	1.8±0.7	2.2±0.9	0.018
FEV1(% pred)	74.2±28.0	67.2±26.8	75.5±27.9	0.144
FVC(L)	2.7±0.9	2.5±0.6	2.8±1.1	0.061
FVC (% pred)	84.8±27.2	79.5±21.1	85.8±25.8	0.180
FEF 25-75(L/s)	2.1±1.5	2.1±1.4	2.3±1.5	0.903
FEF 25-75(% pred)	51.0±31.0	48.3±36.7	50.6±27.8	0.828
FEV1/FVC%	73.0±17.1	67.8±21.0	75.9±12.9	0.074
FEV1/FVC <0.7	28(34%)	13(43%)	15(29%)	0.183
**Health-related quality of life**				
AQLQ symptom	4.6(4.0-6.4)	4.7(4.2-6.4)	4.8(3.8-6.6)	0.865
AQLQ emotional	5.6(1.5-5.0)	5.7(4.0-7.0)	5.7(3.0-7.0)	0.863
AQLQ environmental	3.0(1.5-5.0)	3.0(1.0-4.5)	3.7(1.7-5.7)	0.239
AQLQ activity	5.5(3.9-7.0)	4.9(3.1-6.3)	6.5(5.0-7.0)	0.017*
AQLQ total	4.7(3.7-5.9)	4.6(3.9-5.6)	4.9(3.6-6.2)	0.244
**ACT score**	18.0(13.0-22.0)	18.0(13.0-22.0)	19(14.0-23.0)	0.910

Data are shown as means ± SD or medians (interquartile range), or numbers (percentages of respondents). † Student’s t-test or Mann Whitney test for continuous variables; Pearson chi-square for categorical variablerespondents). † Student´s t-test or Mann Whitney test for continuous variables; Pearson chi-square for categorical variable

Table 2 showed the correlation between the four domains, and the total scores of health-related quality of life and ACT scores. The total score of the AQLQ showed moderate and positive correlation with the ACT scores (rho=0.53 95% CI 0.35, 0.67). A similar relationship was observed across all 4 domains of the AQLQ. The symptoms domain correlated best with the asthma control (rho=0.58; 95% CI 0.42, 0.71) with environmental domain showing the least correlation. The relationship between asthma control and HRQoL became non-significant after adjustment for other factors in environmental domain. ACT alone accounted for 31.0%, 14.7%, 7.5%, 20.3% and 28.5% of variance in symptoms, emotion, environmental, activity limitation subdomain, and total domain respectively in HRQoL. In step 2, the inclusion of psychological distress, allergic rhinitis, % Pred of FEVI and inhaled corticosteroid used also explained an additional non-significant change of 7.3%, 6.9%, 6.5%, 8.3%, except in total domain score, with additional significant variance of 10%. Inclusion of atopy in step 3 also resulted in non-significant variance in the domains except in the emotional domain with an increased variance of 5%, the more likely atopic, the less score of emotional quality of life (Table 3).

**Table 2:** spearman rank correlation coefficient between health-related quality of life and asthma control (n=82)

Health-related Quality of life scores	Asthma control scores (Spearman rho values)	p value
AQLQ symptoms	0.58 (95% CI 0.42-0.71)	<0.001
AQLQ emotional	0.41 (95% CI 0.21-0.58)	<0.001
AQLQ environmental	0.32 (95% CI 0.11-0.51)	0.007
AQLQ activity	0.43 (95% CI 0.24-0.59)	<0.001
AQLQ Total	0.53 (95% CI 0.35-0.67)	<0.001

AQLQ- Asthma Quality of Life Questionnaire, 95% CI- 95% Confidence Interval

**Table 3:** association of atopy and other factors as independent variables with health-related quality of life as dependent variables in hierarchical multiple regression analyses

	Step 1		Step 2				Step 3			
	B	R2	β	R2	ΔR2	p value ΔR2	β	R^2^	ΔR^2^	p value ΔR^2^
**AQLQ Symptom**	0.155**	0.310	0.155**	0.383	0.073	0.069	0.154**	0.398	0.015	0.182
**AQLQ Emotional**	0.126**	0.147	0.120*	0.216	0.069	0.169	0.120*	0.265	0.049	**0.028**
**AQLQ Environment**	0.108*	0.075	0.071	0.140	0.065	0.235	0.070	0.141	0.001	0.751
**AQLQ Activity**	0.148**	0.203	0.119**	0.286	0.083	0.077	0.119**	0.289	0.003	0.590
**AQLQ Total**	0.134**	0.285	0.117**	0.385	0.100	**0.020**	0.117**	0.391	0.006	0.407

Each line refers to results from one hierarchical multiple regression analysis with each domain and total AQLQ as the dependent variable at the 3 steps. Step 1 included Asthma control score. Step 2 included asthma control score, psychological distress, presence of allergic rhinitis, percentage predicted of Forced Expiratory Volume in one second and Inhaled corticosteroid use. Step 3 included all the variables in step 2 and atopic status. β refers to βeta coefficient. * for β value significant with p<0.05 and ** for β value significant with p<0.001. R^2^ refers to variance or coefficient of determination. ΔR^2^ refers to the change in R^2^ between the previous model and the current model. AQLQ: Asthma Quality of Life Questionnaire

## Discussion

Our study revealed that in a clinical sample of adults with asthma, asthma control as measured by ACT was moderately correlated with asthma specific measures of HRQoL using mini AQLQ in all the domains except in the environmental and total scores after adjusting for other clinical factors. In addition, being atopic, contributed uniquely to emotional domain in health-related quality of life Our finding of significant moderate positive correlation between asthma control and health related quality of life gave further support for the association between asthma control and health related quality of life. This is consistent with prior cross-sectional and prospective cohort studies [13,27-29]. In addition, our study also revealed that the symptoms domain correlated most with HRQoL, this may not be surprising as the ACT measures frequency of symptoms (e.g., “How often have you had shortness of breath?”) and functional impairment due to symptoms (e.g., “How much of the time did your asthma keep you from getting as much done at work or at home?”) and HRQoL questionnaire contains 5 questions on symptoms out of the total 15 questions. This may be interpreted as evidence for (cross-sectional) construct validity and consistent with prior study [4].

The findings of this study extend previous works by exploring the unique contribution of atopy to health-related quality of life, interestingly, being atopic contributed significantly to emotional domain in the quality of life after controlling for some covariates that can impact on quality of life. The association between atopic diseases and emotion has been known for some time but whether this represent pathogenetic relevance or an epiphenomenon of chronic inflammatory disease is currently being debated [30]. Interestingly, some studies have reported that there are connections between allergy-related inflammation and emotional disorders, but the underlying mechanism of this association remains unclear. The possible underlying mechanism could be that pro-inflammatory cytokines involved in the pathogenesis of allergy-related diseases, such as interleukin (IL)-6, tumor necrosis factor-alpha (TNF-Þ), IL-10, and monocyte chemoattractant protein-1/CCL2 might be well associated with emotional disorders [31]. Our study and other studies [18,31,32] suggest sensitization to allergen may be important contributor to perceived impact of asthma on emotional wellbeing. This finding would encourage health professionals to comprehensively assess and manage atopic asthmatics. Assessing their emotional condition and managing it appropriately could translate to a better quality of life. Additional and notable finding in our study is the difference that was observed in the activity domain of health-related quality of life between atopic and non-atopic asthmatics, although data that examined specifically the relation between atopic status of asthma and HRQoL are scarce. Kannan *et al*. [33] in their study of elderly asthmatics reported that atopy was significantly associated with higher activity domain scores in consonance with our finding. In contrast, the study by Coban and Aydemir did report higher value for non-atopic compared to atopic asthmatics, in both the total score and specific domains, however, the difference is not significant [16]. Reasons for differences in asthma-related quality of life based on atopic status have not been fully explained. Although it might be expected that atopic asthmatics should have lower quality of life scores because exposure to allergens is known cause of asthma exacerbation and sustained symptoms in atopic asthmatics. In our study, atopic asthmatics had a better quality of life in the activity domain; a possible explanation could be that atopic individuals are younger and therefore more active.

The distinct strength of this study is the adjustment for known covariates that are known to affect asthma related quality of life. These co-variables were chosen a-priori based on previous studies. We also demonstrated atopy by skin prick test. According to GINA guideline, skin prick test is simple and rapid to perform with a high sensitivity and it is not less reliable than specific IgE measurement. It is essential to recognize some limitations to this present study. First, the study population, may not be representative of the general population of adults with asthma as they are largely drawn from hospital-based sample and most asthmatics may not attend routine clinical check-up in our environment. However, recruitment was over a year period, so that we recruited as many asthmatics as possible to improve the generalizability of our data. Second, we did not assess for baseline asthma severity, although this may appear to confound the result. The ultimate goal of asthma treatment is to achieve control and this can be achieved irrespective of the baseline asthma severity. More so, Lavoie *et al*. reported that the relationship between asthma control, health-related quality of life and psychological distress may be independent of objective asthma severity and prescribed treatment level [34]. Third, our sample size was not specifically designed to look for the difference between atopic and non-atopic asthmatics. This may not be practicable as we do not have the database of atopic asthmatics or non-atopic asthmatics. However, our data provides preliminary report on the contribution of atopy to poorer quality of life in emotional domain. Furthermore, there would be need for more studies specifically designed to assess the difference between atopic and non-atopic asthmatics.

## Conclusion

In summary, our data suggests that asthma control as measured by ACT, correlates moderately with health-related quality of life except in environmental domain and that atopy as measured by skin prick test contributed to quality of life in emotional domain. Improving health-related quality of life is a major emphasis in chronic diseases such as asthma, so health professionals may need to pay a more detailed attention to atopic asthmatics, and probably explore the impact of their disease on their emotions.

### What is known about this topic

- Asthma control contributes to asthma specific health-related quality of life;
- In asthmatics, some clinical, demographical and psychological variables are also associated with health-related quality of life.

### What this study adds

- After controlling for known factors that can affect quality of life in asthmatics, asthma control is still associated with health-related quality of life except in environmental domain;
- Atopy made a unique significant contribution to emotional domain of health-related quality of life.
